# Jetting Dynamics of Burning Gel Fuel Droplets

**DOI:** 10.3390/gels8120781

**Published:** 2022-11-29

**Authors:** Janmejai Sharma, Ankur Miglani, Jerin John, Purushothaman Nandagopalan, Javed Shaikh, Pavan Kumar Kankar

**Affiliations:** 1 Microfluidics and Droplet Dynamics Lab, Department of Mechanical Engineering, Indian Institute of Technology, Indore 453552, Madhya Pradesh, India; 2 Department of Aerospace Engineering, Korea Advanced Institute of Science and Technology, 291, Daehak-ro, Yuseong-gu, Daejeon 34141, Republic of Korea; 3 Department of Aerospace Engineering, Hindustan University, Chennai 603103, Tamil Nadu, India; 4 Intel Corporation, Bengaluru 560035, Karnataka, India

**Keywords:** gel fuels, ethanol, organic gellants, droplet combustion, hybrid rocket

## Abstract

Jetting in burning gel fuel droplets is an important process which, in addition to pure vaporization, enables the convective transport of unreacted fuel vapors from the droplet interior to the flame envelope. This aids in accelerating the fuel efflux and enhancing the mixing of the gas phase, which improves the droplet burn rates. In this study, Schlieren imaging was used to characterize different jetting dynamics that govern the combustion behavior of organic-gellant-laden ethanol gel fuel droplets. To initiate jetting, the gellant shell of the burning gel fuel droplet was subjected to either oscillatory bursting or isolated bursting, or both. However, irrespective of the jetting mode, the jets interacted with the flame envelope in one of three possible ways. Based on the velocity and the degree to which a jet disrupts the flame envelope, it is classified as either a flame distortion, a fire ball outside the flame or a pin hole jet (localized flame extinction), where the pin hole jets have the highest velocity (1000–1550 mm/s), while the flame distortion events have the lowest velocity (500–870 mm/s). Subsequently, the relative number of the three types of jetting events during the droplet lifetime was analyzed as a function of the type of organic gellant. It was demonstrated that the combustion behavior of gel fuels (hydroxypropyl methylcellulose: HPMC at 3 wt.%) that tend to form thin-weak-flexible shells is dominated by low-velocity flame distortion events, while the gel fuels (methylcellulose: MC at 9 wt.%) that facilitate the formation of thick-strong-rigid shells are governed by high-velocity fire ball and pin hole jets. Overall, this study provides critical insights into the jetting behavior and its characterization, which can help us to tune the droplet gasification and the gas phase mixing to achieve an effective combustion control strategy for gel fuels.

## 1. Introduction

The realization of future rocket motor engines will depend on their adaptability to next-generation fuels that are environmentally friendly, operationally safe, easy to process, and reliable [1,2,3]. In this regard, gel fuels have attracted significant interest as high-performance alternatives to the traditional rocket fuels. This is due to their improved rheological and physical properties that bring together the key functional traits of solid and liquid fuels, as demonstrated by Hodge K. et al. [4]. For example, gel fuels behave in a solid-like manner at low shear, which mitigates the chances of an accidental hazard due to a leak or spillage, especially in the case of hypergolic and toxic fuels, as per the findings of Ciezki H.K. et al. [5]. During storage, the high viscosity allows the energetic nanoparticles to be suspended stably in the fuel matrix, which aids in the enhancement of the energy density of the fuel and the engine’s specific impulse, as per the studies of Rahimi S and Natan B [6]. At the other end, the liquid-like behavior at high-shear stresses due to the shear-thinning property enables easier pumping and atomization when the gels are forced through injectors at high pressures [6,7,8,9,10,11]. These liquid-like characteristics can provide critical functionalities, such as re-ignitability and thrust modulation in rocket engines [6,7,8,9,10,11].

While gel fuels are an attractive alternative to their solid and liquid counterparts, their implementation has been limited due to the lack of understanding of their complex combustion behavior, which is highlighted by the disruptive vapor-jetting events. The jetting of fuel vapors, or simply jetting, as proposed by Solomon Y et al. [12,13], refers to the discharge of unreacted fuel vapor from the droplet interior, which occurs aperiodically and asymmetrically during the droplet lifetime. In the existing literature, as examined by Mishra D.P. et al. [14,15], the term *microexplosion* is often used in place of *jetting* to characterize the disruptive combustion behavior of gel fuel droplets. However, microexplosion and jetting represent two distinct types of disruption events, which have completely different initiation mechanisms and impacts on the droplet. If the explosion is confined to a segment of the parent droplet and is of a lower intensity, it is termed as puffing, which is characterized by the blowing out of vapor, along with a stream of fine droplets. Microexplosion was first reported by Law [16,17,18] in the context of multiphase multicomponent fuel droplets, corresponding to an abrupt catastrophic fragmentation of the droplet due to the rapid gasification of the high-volatility species entrapped in the droplet core. In multicomponent droplets possessing species with a high volatility differential, the lower-boiling-point (or the higher-volatility species) species becomes diffusionally entrapped in the core, while the lower-volatility-species accumulates on the surface and governs the droplet surface temperature. With the increasing droplet temperature, the entrapped species is superheated to its homogenous boiling limit and undergoes explosive boiling, which is accompanied by a rapid pressure rise. Since homogenous boiling is accompanied by a sudden release of a large amount of stored superheat, this event (termed as microexplosion) shatters the entire droplet into smaller fragments and marks an end to the droplet lifecycle. In contrast to microexplosion, jetting events are unique to gel fuels and occur continuously during their lifetime, which features the initiation, coalescence, growth, and eventual collapse of bubbles to release the jets of unreacted fuel vapors [12,13,19,20]. The continuous formation and collapse of multiple bubbles are representative of heterogeneous boiling. Because heterogeneous boiling is characterized by a low degree of stored superheat compared to homogenous boiling, the jetting events triggered by heterogenous boiling have a visibly low intensity compared to a microexplosion event [19,21,22].

With respect to the disruptive events that are triggered by heterogeneous boiling, the jetting of the unreacted fuel vapors is like the bubble ejection events that govern the combustion behavior of nanofluid fuel droplets [21,22,23,24,25]. However, in terms of the mass, momentum, and energy transport processes that control their behavior, starting with initiation and ending with their termination, and their subsequent effects on droplet combustion, these are widely different disruptive events. First, in nanoparticle-laden fuel droplets, the particles agglomerate either via orthokinetic or perikinetic mechanisms to form micrometer-sized aggregates that behave as *sites* of bubble nucleation, as reported by Miglani A et al. [23]. In contrast, when burning gel fuel droplets, the inner surface of the gellant shell acts as a surface for bubble nucleation. Secondly, in nanoparticle-laden fuel droplets, the pressure upsurge due to internal boiling is released via the rupture of the shell formed of agglomerated nanoparticles. The shell rupture is accompanied by the formation of *child droplets* that break off from the parent nanofuel droplet [23,24]. These child droplets may further undergo microexplosion to form more child droplets [26,27,28] In comparison, the rupture of the gellant shell in the case of gel fuel droplets does not form any *child droplets*. Instead, the internal pressure build-up is released by the jetting of unreacted fuel vapors via the ruptured location. Details of the lifecycle of a vapor jet and the mechanisms governing it can be found in Miglani A. et al. [19]. Thirdly, the shell formation process, in the case of nanoparticle-laden droplets, is controlled by the interplay between two competing mechanisms, namely, particle agglomeration and the secondary atomization of droplet, both of which change the mass fraction of NPs within the droplet at any time instant. The former helps to build the shell via particle agglomeration, while the latter impedes shell formation directly by rupturing it and indirectly by depleting the population of NPs through atomization, as reported by Miglani A. and Basu S. [21]. In contrast, the shell formation, in the case of gel fuel droplets, occurs via the phase separation of the gellant [12,13,29,30,31,32,33] from the mixture of the base fuel, the gellant, and the gellant solvent. In this case, the entire mass of the gellant is retained by the droplet until the end of the droplet lifetime, when the shell carbonizes and is consumed. Fourthly, in nanoparticle-laden droplets, the child droplets resulting from secondary atomization and their subsequent fragmentation (tertiary atomization) aids in improving the droplet burn rate by enhancing the net surface area and dispersing the fuel charge evenly, as reported by Rao D.C. et al. [27]. Furthermore, the child droplets behave as nanoparticle carriers and transport them to the flame, where the particle ignites and releases energy [22,25]. In contrast, the jetting allows the unreacted fuel vapors to be transported advectively from the droplet to the flame envelope. This helps to improve the droplet burn rate by increasing the fuel mass flux, in addition to pure vaporization. Since the droplet burn rates are significantly influenced by the type of jetting events and variations in their number density during the droplet lifetime, it is imperative to characterize the jetting events. This is particularly crucial for rocket motor applications utilizing gel propellants, which exhibit higher ignition delays and low burning rates due to their inherently high heat of vaporization [33]. To this end, a key objective of this study is to characterize the jetting events and investigate their effects on the droplet flame as a function of the nature of the gellant shell.

## 2. Results and Discussion

**Figure 1:**
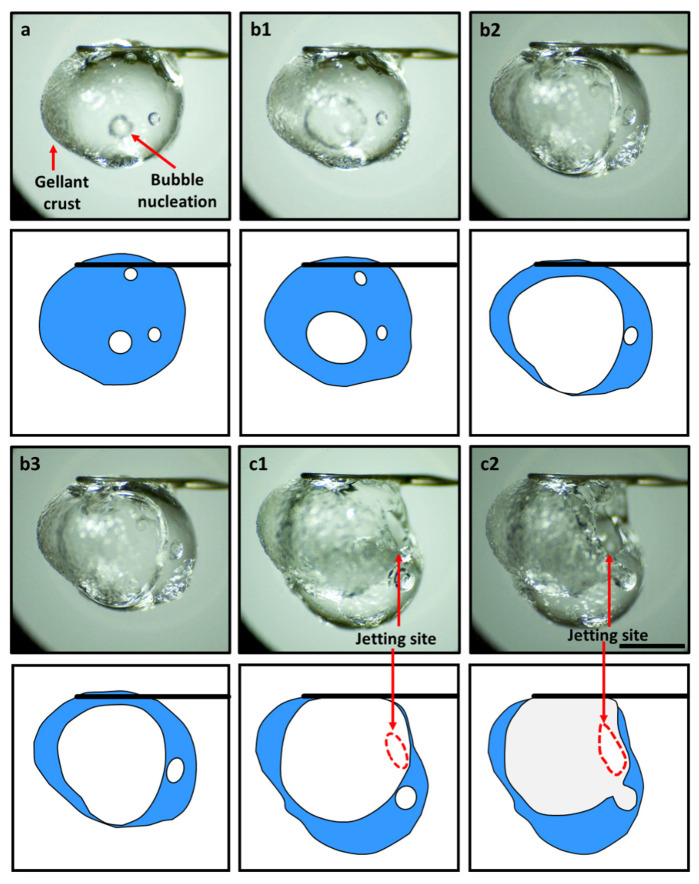
High-magnification images (**top**) and the accompanying schematic representations (**bottom**) of the precursor events that lead to the initiation of jets. (**a**) Formation of gellant crust/shell and bubble nucleation, (**b**) bubble growth and droplet expansion (**b1**: initial bubble growth, **b2**: bubble expansion occupying large droplet vapor fraction, and **b3**: swelled droplet state prior to rupture, and (**c**) shell rupture and jetting of unreacted fuel vapors (**c1**: formation of rupture site, and **c2**: expansion of rupture site due to viscoelastic nature of the gellant shell. The scale equals 1 mm.

**Figure 2:**
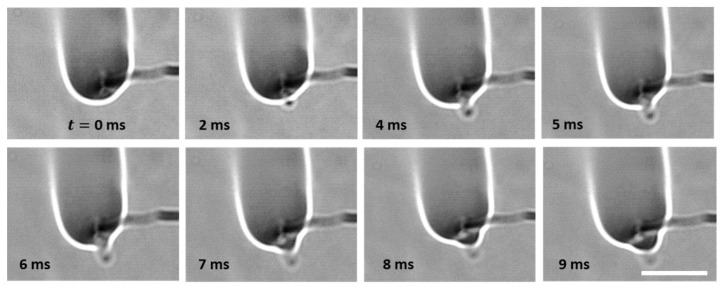
High-speed images showing a representative jetting event and its interaction with the droplet flame envelope. The scale equals 10 mm.

The disruptive burning behavior of gel fuel droplets is characterized by the jetting of unreacted fuel vapors [12,13,14,15] that occur unevenly during the droplet lifetime. A typical jet is initiated through a three-step process that occurs inside the droplet [12,13,14,15], as shown in Figure 1. First, during the transient heat-up period, the gellant phase separates from the base fuel/gellant solvent, which results in the formation of a gellant crust near the droplet surface. Second, the trapped fuel (inside the droplet) undergoes boiling. Third, the gellant shell bursts due to internal pressure build-up and jetting of the unreacted fuel vapors (across the droplet–gas interface). In this way, the pressure energy within the bubble is converted to the kinetic energy of the jet, which is released via the rupture site on the droplet shell. Subsequently, the jet travels to the flame envelope, where it interacts with the flame and disturbs the symmetric tear-drop shape of the diffusion flame. Figure 2 shows an example of a typical jetting event and its interaction with the flame during the combustion of an HPMC 3 ethanol gel droplet. The entry of the fuel jet into the flame envelope is visible at 2 ms. At 4 ms, the jet causes localized flame extinction to occur, as is evident from the broken flame front. Subsequently, the flame begins to propagate around the jet of the unreacted fuel vapors and reconnects at 9 ms. Note that in addition to pure vaporization, the jetting enables the advective efflux of the unreacted fuel vapors from the droplet and, therefore, jetting represents a key mechanism of the mass transport of fuel vapors that have the potential to improve the droplet gasification rate significantly [34]. This is crucial for gellant-based fuels because they tend to have high heats of formation and, hence, low gasification rates [33]. Therefore, in this section, the different types of jetting events are characterized in terms of their average velocity and their impacts on the flame envelope are analyzed first. Second, the effects of the gellant type and, hence, the nature of the gellant shell on the number and average velocity of the jetting events are presented.

### 2.1. Jetting Modes

**Figure 3:**
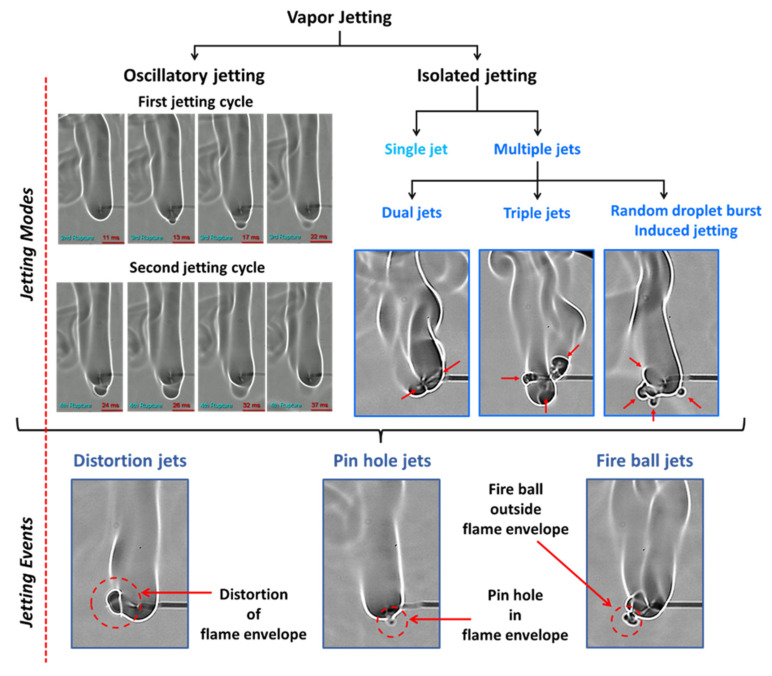
The jetting modes and events that govern the combustion behavior of organic-gellant-laden gel fuel droplets.

**Figure 4:**
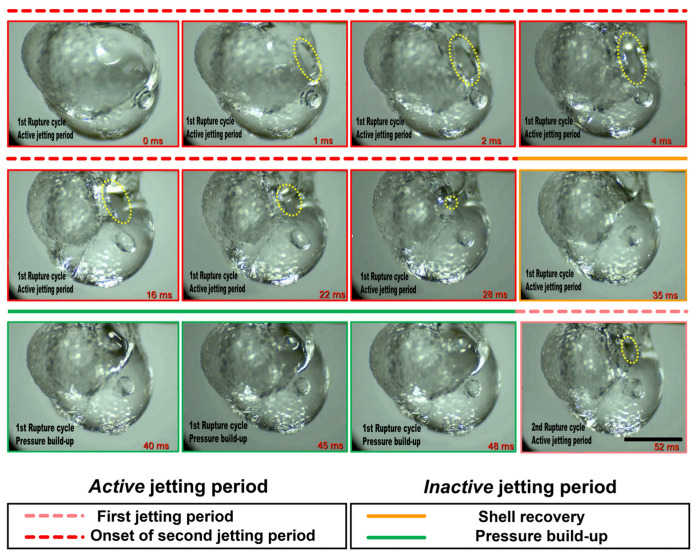
Dynamic rupture sequence of a combusting gel fuel droplet undergoing oscillatory bursting. The scale equals 1 mm.

**Figure 5:**
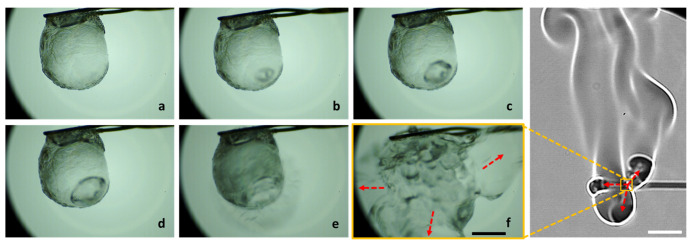
High-magnification images showing random bursting of a burning gel fuel droplet. (**a**).Gel droplet with the gellant shell. (**b**) Bubble nucleation on the inside surface of gellant shell. (**c**,**d**) Bubble growth inside the droplet. (**e**) Initiation of droplet burst at a single rupture site. (**f**) Droplet bursting at multiple random locations. The resultant jetting and its interaction with the flame envelope is shown on the right. The red arrows indicate the directions of jetting at the droplet and flame scale respectively. The scale equals 1 mm for the droplet images and 10 mm for the flame image.

Depending on the interplay between the degree of pressure rise within the droplet and the ability of the shell to restrain this pressure due to its rheo-physical properties, the droplet may exhibit different jetting modes, namely, either isolated jetting or oscillatory jetting, or both (see Figure 3). Oscillatory jetting features the periodic release of jets from the same region of the droplet and occurs in gel fuels that tend to form viscoelastic shells that are weak but flexible [19]. This allows the shells to undergo oscillatory bursting cycles featuring shell rupture and recovery. As shown in Figure 4, a single bursting cycle comprises of two phases: first, the active jetting period, where the jets are initiated due to shell rupture and the jetting continues until the shell recovers, and second, the inactive jetting period, where the shell recovers and, subsequently, the pressure builds up to initiate the next bursting cycle. In this way, the oscillatory bursting cycles of the shell result in oscillatory jetting. In HPMC-based gel fuel droplets, which form a weak-flexible viscoelastic shell, oscillatory jetting occurs over ~10–70% of the droplet lifetime, depending on the gellant concentration [19]. Therefore, oscillatory jetting is a key jetting mode that governs the droplet burn rate via the efflux of unreacted fuel vapors. In contrast, the gel fuels that tend to form strong-rigid shells feature isolated jetting [19]. The effect of the gellant type on the nature of the shell and its effect on the jetting behavior are discussed later in Section 2.3. The isolated jetting mode is characterized by the release of individual jets, which may occur as either single isolated jets or as multiple simultaneous jets at different spatial locations across the droplet surface. Multiple jets occur either when the droplet shell ruptures at more than one location or in a random event where the shell is blown apart due to a transient pressure upsurge, as shown in Figure 5. In both these cases, the ruptured shell forms multiple jetting sites, which results in the large-scale distortion of the flame envelope.

### 2.2. Jetting Events

While the jetting modes are governed by competition between the internal boiling activity that tends to build up pressure (due to bubble growth) and the ability of the viscoelastic shell to contain this pressure rise, the resulting individual jets interact with the flame in one of the following ways: first, the jet distorts the flame envelope without breaking it; second, the jet breaks and moves past the flame envelope, where the unreacted fuel vapors ignite to form a fire ball outside the droplet flame envelope; and third, the jet punctures the flame envelope and creates a pin hole in the flame envelope. Accordingly, the three distinct types of jetting events are termed as flame distortion, a fire ball outside the flame, and pin hole ejections, respectively, which are shown at the bottom of Figure 3. These jetting events are characterized based on their average velocity and their effects on the flame envelope in terms of the temporal variation in the flame perimeter during the jet lifetime. Table 1 details the average velocities of these three events, which indicate that pin hole jets have the highest speed, while flame distortion events have the lowest speed.

**Figure 6:**
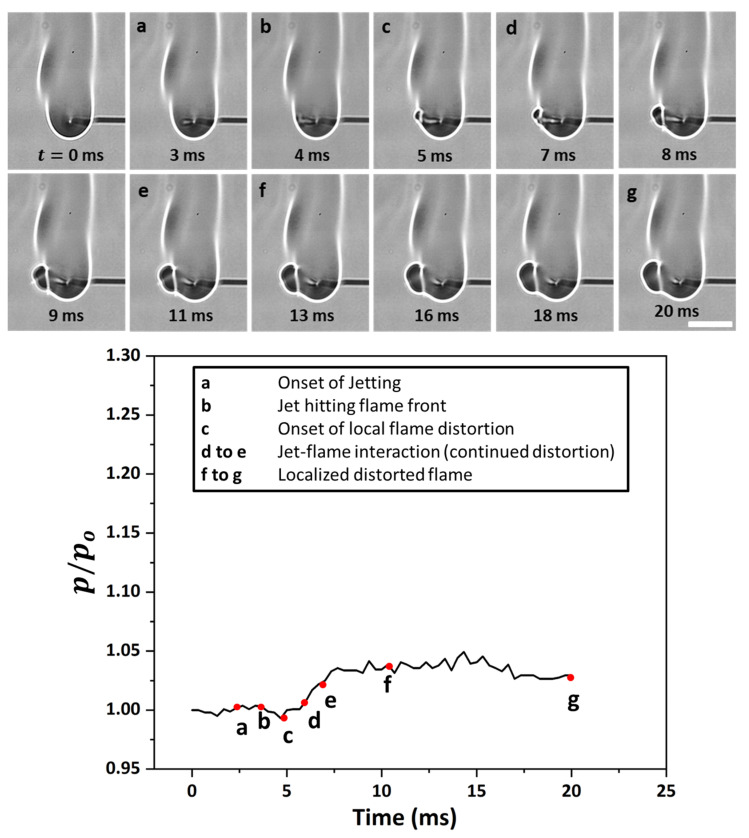
(**Top**) Time-frozen snapshots of a jet causing flame front distortion. The scale equals 12 mm. (**Bottom**) Temporal variation in the normalized flame perimeter characterizing the flame envelope disruption.

**Figure 7:**
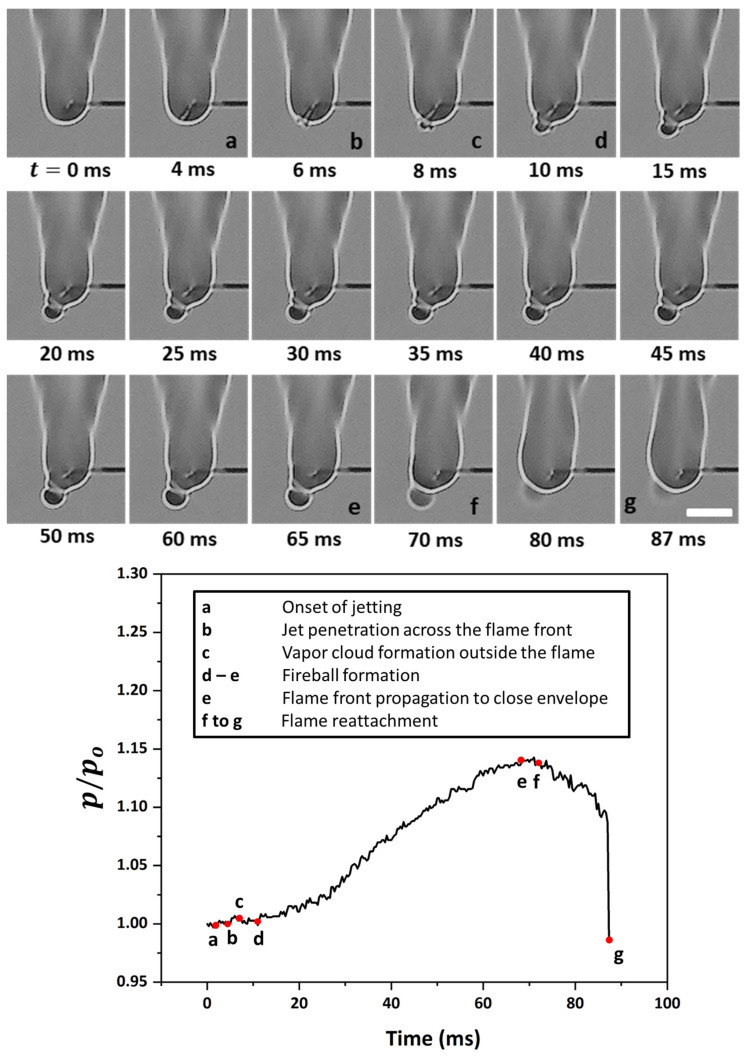
(**Top**) Time-frozen snapshots of a jet forming a fire ball outside the flame envelope. The scale equals 5 mm. (**Bottom**) Temporal variation in the normalized flame perimeter characterizing the flame envelope disruption.

**Figure 8:**
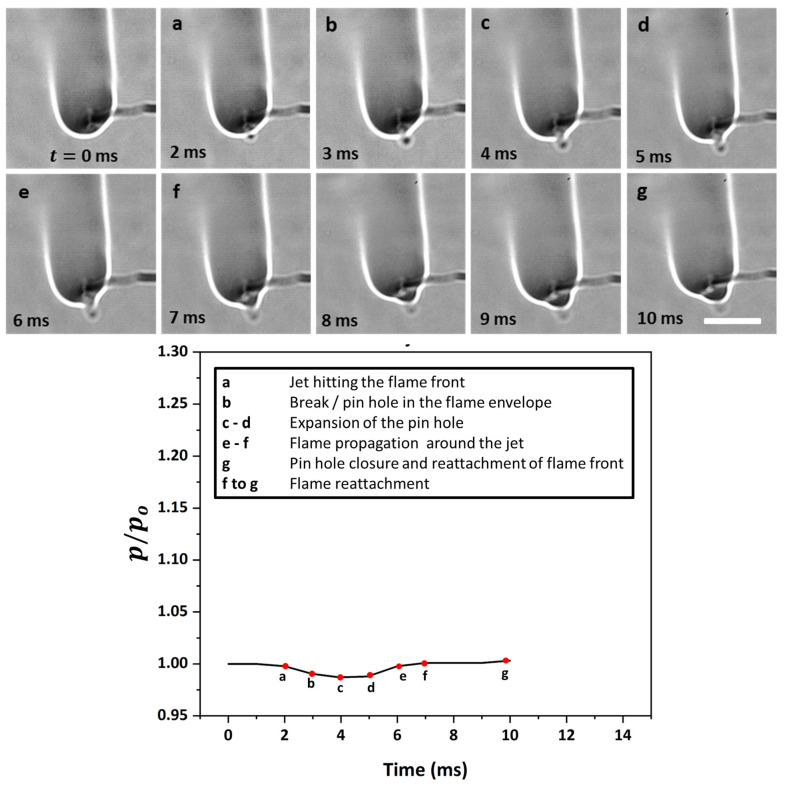
(**Top**) Time-frozen snapshots of a jet breaking the flame front. The scale equals 10 mm. (**Bottom**) Temporal variation in the normalized flame perimeter characterizing the flame envelope disruption.

Figure 6 shows the time sequence images of a flame distortion event, where the jet emanates at approximately 180° from the droplet at time t= 0 ms, travels to the flame front, and hits the envelope at 5 ms. This jet induces distortion in the flame envelope due to its radial momentum and its expansion as it hits the high-temperature flame envelope. For such events, the average velocities are low enough that the flame curvature undergoes distortion but remains intact, i.e., it does not undergo localized extinction. The fact that the flame remains distorted for a long enough period instead of being spontaneously restored to its initial shape demonstrates that the evaporated fuel is discharged continuously via the ruptured shell. In this representative flame distortion event, the speed is ~884 mm/s, and a noticeable distortion is observed in the flame front, as seen by a change in its perimeter (see the bottom plot in Figure 6). Note that while the maximum variation in the normalized perimeter is just 5%, the perimeter of the flame front can change significantly during several other flame distortion events that occur in droplet’s lifetime. Next, Figure 7 shows the time-frozen snapshots of the jetting event that leads to fire ball formation outside the flame envelope. Fire ball formation involves a two-step process: first, the jet breaks the flame front, and second, before the flame propagates around the jet and repairs this break in the flame envelope, the unburned fuel vapors that are the outside the flame envelope begin to react to form a fire ball, which grows over time. Here, the first step of the break in the flame occurs rapidly in ~6 ms, with a negligible change of less than 2% in the flame perimeter. In contrast, the second step of fire ball formation and growth occurs over a longer timescale of ~60 ms and features a significant increase in the flame perimeter by ~15% before the flame envelope is restored to its initial steady-state value. Therefore, the fireball events tend to form a separate flame front away from the parent flame, and the effect of these events lasts for a longer time, as seen by the time scale O (70 ms). The speed of the fire ball event shown in Figure 7 is 1012 mm/s. The jets that tend to break the flame front are characterized by high speeds, which are higher compared to both flame distortion and the fire ball events. Figure 8 shows a typical pin hole jetting event with a speed of 1356 mm/s, which hits and breaks the flame front just ~2 ms after its initiation. Subsequently, the flame propagates around the vapor jet to repair the break in the flame envelope and restores the complete envelope at 10 ms. This indicates that the pin hole jets are the fastest (and, hence, the shortest jetting events in terms of the timescale) and, therefore, have a negligible impact on the flame perimeter, as shown in the bottom plot of Figure 8. These events are caused by a high pressure build up within the droplet and its subsequent release from a tiny rupture site (in the form of either a small crack or a pin hole) in the shell in the form of a high-velocity jet. The formation of localized tiny rupture sites is characteristic of gellants that tend to form strong-rigid shells, which have a high tensile strength and can sustain high pressures before undergoing rupture. The fact that the flame perimeter reduces slightly as soon as a pin hole jet is initiated and remains at the reduced value for the entire jetting duration indicates that the droplet shell sustains a high pressure, which is released suddenly in a short duration. This pressure release is accompanied by droplet shrinkage and, hence, a reduction in the flame size. Additionally, the high speed of the pin hole jet ensures that the time duration for the jet’s interaction with the high-temperature flame envelope is minimal, which prevents jet expansion and, hence, its large-scale distortion. Note that compared to the pin hole jets, where the jet–flame interaction occurs over short timescales of <10 ms, the flame distortion events occur for a substantial duration ~O (100 ms), which allows the jet to distort the flame structure significantly.

**Figure 9:**
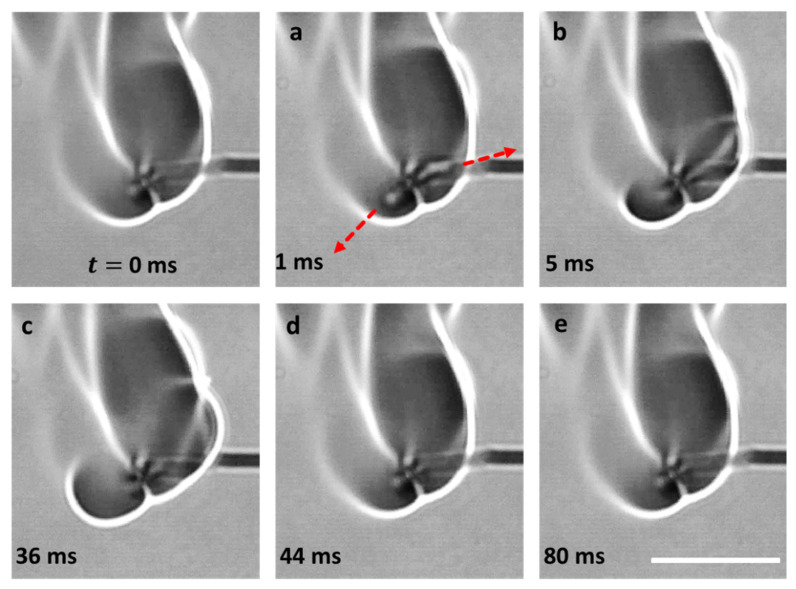

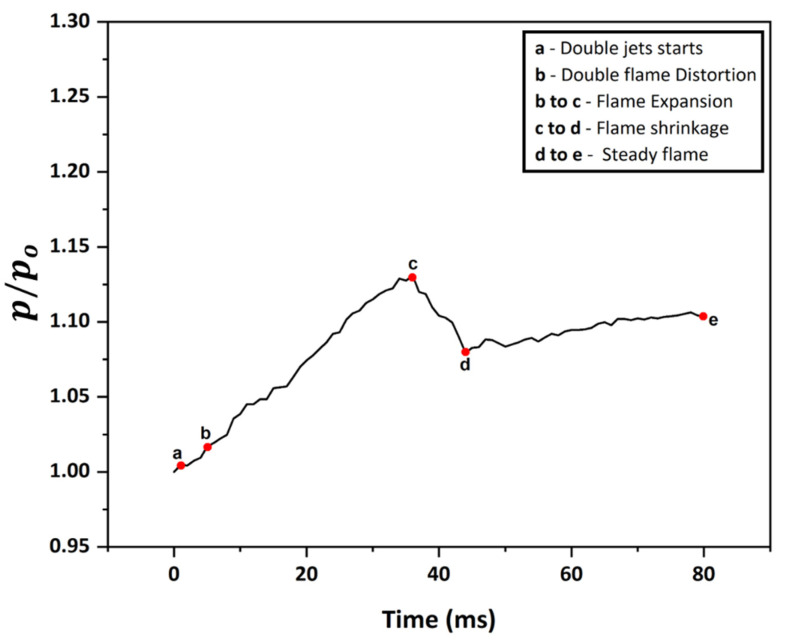
(**Top**) Time-frozen snapshots of a double-jet event comprising of two flame distortion events aligned at two different angular locations. The scale equals 15 mm. (**Bottom**) Temporal variation in the normalized flame perimeter characterizing the flame envelope disruption.

**Figure 10:**
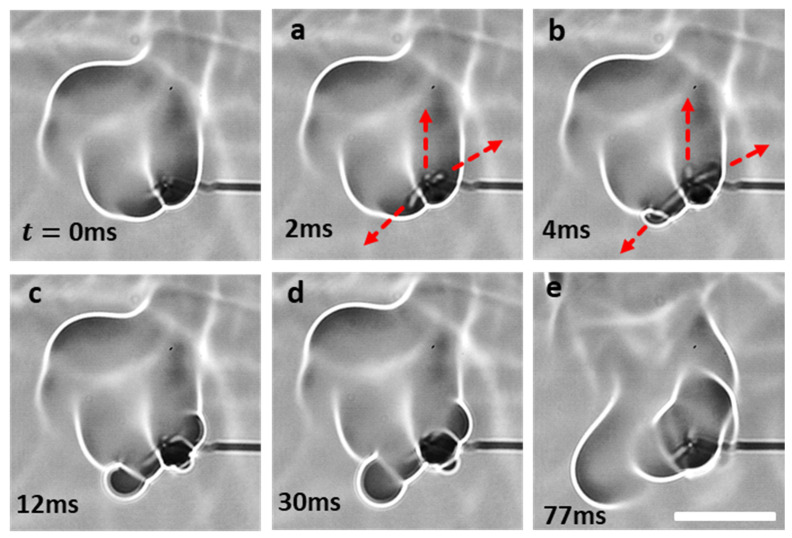

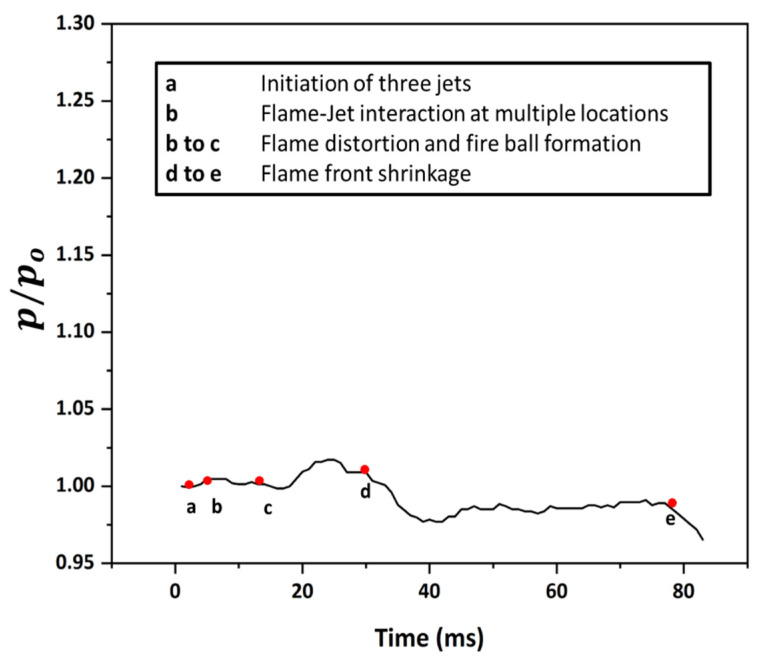
(**Top**) Time-frozen snapshots of a triple-jet event comprising of two fire balls and one flame distortion event, each aligned at a different angular location. The scale equals 20 mm. (**Bottom**) Temporal variation in the normalized flame perimeter characterizing the flame envelope disruption.

**Figure 11:**
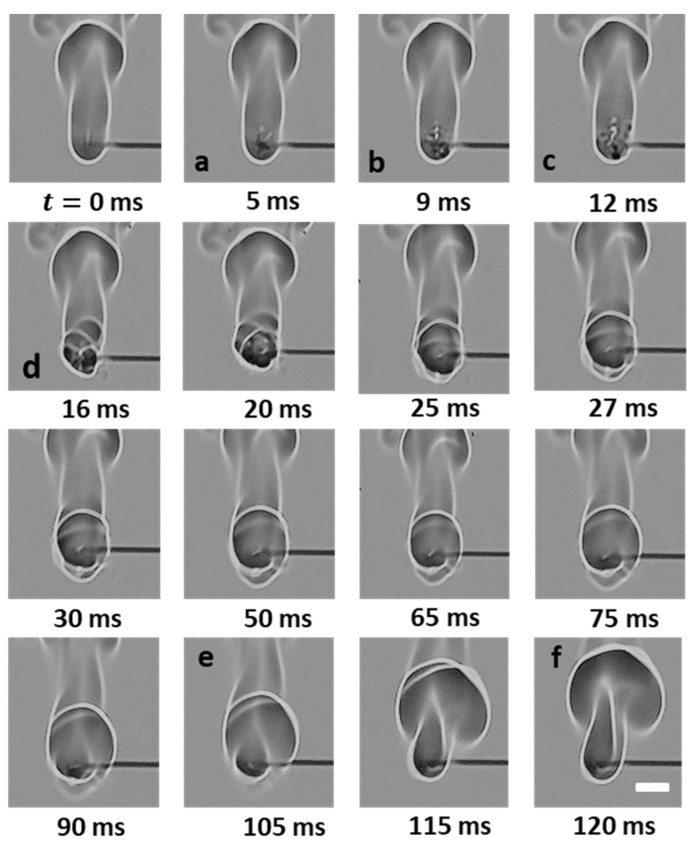
Time-frozen snapshots of multiple jetting events caused by the random bursting of a gel fuel droplet. The scale equals 5 mm.

**Figure 12:**
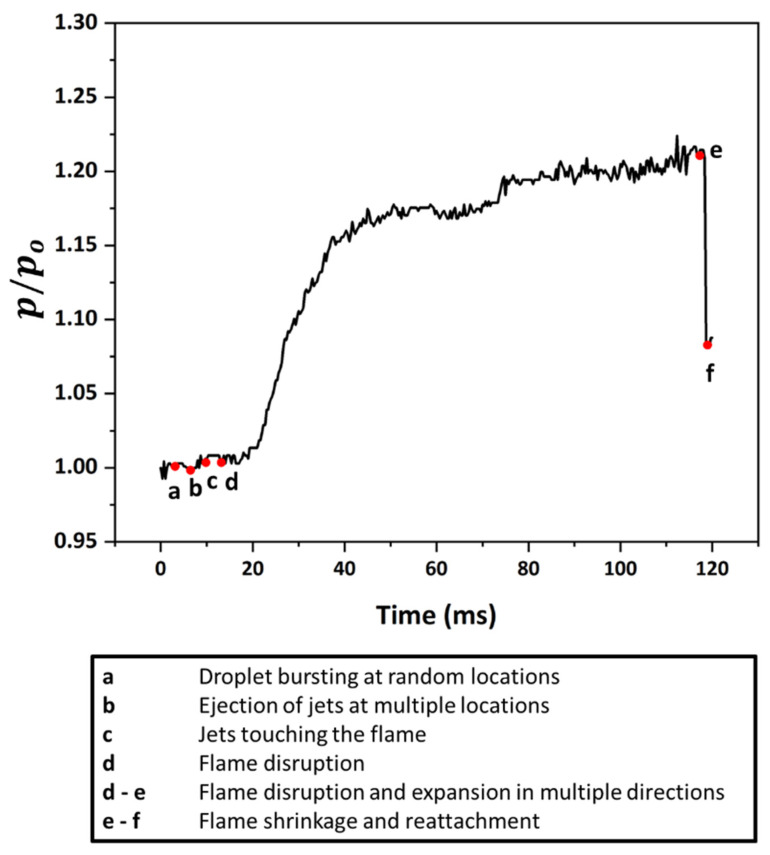
Temporal variation in the normalized flame perimeter resulting from the disruption caused by the random bursting event with multiple jets.

Due to the inhomogeneous structure of the gellant shell [19], the shell may rupture at more than one location, and therefore, the three types of jetting events may not occur individually, i.e., only one at a time. Instead, multiple events may either overlap or occur simultaneously. This is evident in Figure 9 and Figure 10, which show the occurrence of two and three simultaneous jetting events, respectively. These are termed as double and triple jetting events, respectively. Depending on the shell structure and the size of the rupture hole, any combination of flame distortion, pin hole, and fire ball events can occur during a multiple jetting event. For instance, the double-jet event in Figure 9 constitutes a set of two flame distortion events occurring at 55° and 235° at speeds of 927 mm/s and 960 mm/s, respectively. However, the triple-jet event shown in Figure 10 constitutes a set of two fire ball events and one flame distortion event aligned at 38°, 178°, and 262°, respectively and their corresponding velocities are 1415 mm/s, 1562 mm/s, and 1121 mm/s, respectively. Due to interactions of multiple different types of jetting events with the flame, it is expected that the flame envelope undergoes random shape changes over time. This is corroborated by the non-monotonic trend of the normalized flame perimeter over time (see Figure 9 and Figure 10). Note that a drop in the flame perimeter is observed between 36 and 44 ms in the case of a double-jet event and between 30 and 77 ms for a triple jetting event. This is due to the termination of one of the flame distortion events in this timeframe in both the cases, which reduces the degree of distortion in the flame envelope. The occurrence of multiple jetting events is also observed in a random event when the droplet undergoes sudden bursting, as shown in Figure 11. This random bursting event occurs following the droplet’s transient heat-up period, when the bubble nucleates on the shell inner surface and expands rapidly in a short time (<2 ms), causing the shell to rupture at multiple sites. Since the sudden bursting of the droplet is an uncertain event and occurs rapidly with the formation of multiple rupture sites (and, hence, multiple jets), it disrupts the flame randomly. This is primarily because the jets have no specific spatial orientation, and any combination of the three types of jetting events may occur. This is evident from the non-monotonic variation in the flame perimeter over time (see Figure 12), which shows that the flame perimeter remains nearly constant until 20 ms, then increases rapidly from 20 to 40 ms by ~15%, then increases gradually from 40 to 110 ms by ~5%, and finally exhibits a sharp drop of ~13% in less than 5 ms. In comparison to the other flame disruption events, the random bursting event has a lasting impact on the flame structure, as the flame remains distorted for longer timescales on the order of ~O (200 ms). It is important to note that even though the jetting event has finished, the flame may continue to be disrupted due to the expansion of the unreacted fuel vapors and their local interaction with the flame envelope. Subsequently, as the flame envelope stabilizes and reaches a steady state, its shape and perimeter may not be the same in this new state as they were before the commencement of a flame disruption event.

### 2.3. Effect of the Gellant Type on the Jetting Events

**Figure 13:**
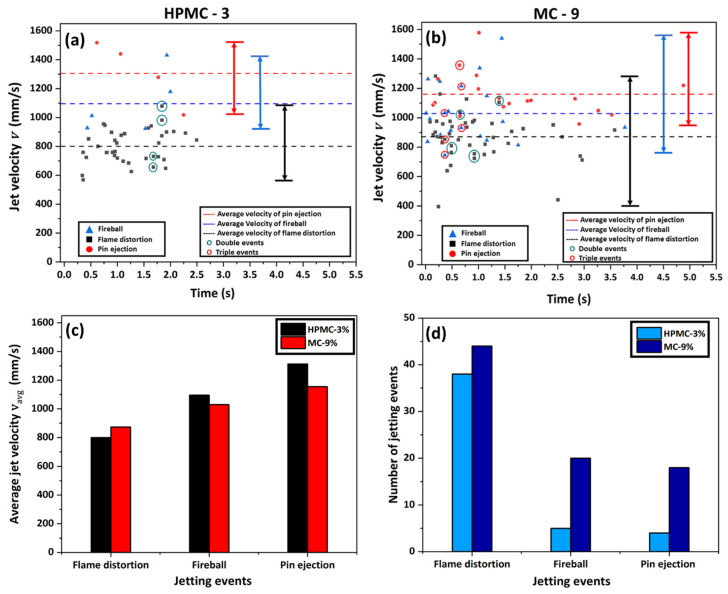
(**Top**) The variation in the jet velocities as a function of time during the combustion of a gel fuel droplet: (**a**) HPMC-3 and (**b**) MC-9. The range of the velocities (vertical double-headed arrows) is shown for all three jetting events and are color-coded as black for the flame distortion events, blue for fireball events, and red for pin ejection events, alongside their average values (horizontal dotted lines) to show the range over which the jet velocities are distributed. (**Bottom**) Histograms showing a one-to-one comparison of the variation in the average velocities of three types of jetting events for both the gel fuels (**c**). The number of each type of jetting event (flame distortion, pin hole ejection, and fire ball) for both the gel fuels (**d**).

Figure 13 shows a comparison of the temporal variation in the jetting velocity of the burning gel fuel droplets of HPMC-3 versus MC-9 for six experimental runs. It is evident that for both types of gel fuels, the average velocity is highest in the case of the pin hole jets and lowest in the case of flame distortion events. In addition, within the experimental uncertainty, the average velocity of the three types of jetting events is the same for both the gel fuels (see Table 1). While the average jetting velocities are nearly same (Figure 13c), the number of jetting events during the droplet lifetime are significantly higher in the case of the MC-gellant-based gel fuel droplets. The HPMC-3 fuel tends to form flame distortions (~500–870 mm/s) in substantial numbers in comparisosn to pin ejection (~1000–1550 mm/s) disruptions and fireballs outside the flame (~800–1530 mm/s). In the case of MC-9, along with flame distortions, pin ejections and fireballs outside the flame are found to be in abundance. However, from Figure 13a,b, it can be observed that the range of the velocity distribution of each of the flame disruption events differs for both fuels. Therefore, it is worth mentioning that the velocities depicted in Figure 13a,b correspond to the interaction of the jets with the flame, irrespective of the magnitude of the velocity. Moreover, the number of flame distortion, fire ball, and pin hole jets in the MC-based gel fuel droplet are ~1.15 times, 4 times, and 4.5 times the number of corresponding events in the case of the HPMC-based fuel droplet (Figure 13d). It is also apparent from the bottom plot in Figure 13d that the jetting behavior of the HPMC-based gel fuel droplets is dominated by the flame distortion events, which are approximately 4 times more frequent compared to the high-velocity fire ball and the pin hole jetting events. In contrast, in the case of the MC-based gel fuel droplets, the number of flame distortion events is comparable to the number of fire balls and pin hole ejections.

**Figure 14:**
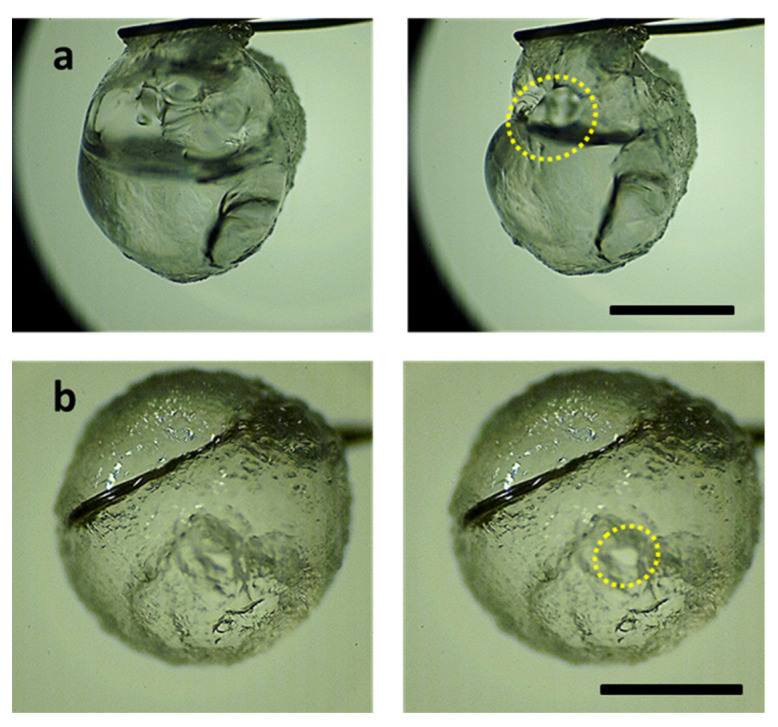
Representative images of rupture site formation during a jetting event: (**a**) HPMC-3, (**b**) MC-9. The scale equals 1 mm.

Furthermore, for the MC-based droplets, the number of double and triple jetting events, together, is more than twice the number of corresponding events in the case of the HPMC-based droplets. In summary, jetting in HPMC-based fuels is governed by low-velocity flame distortion events, while jetting in MC-based gel fuel droplets is significantly influenced by high-velocity fire balls and pin hole jets. The difference observed in the jetting behavior of the HPMC- versus the MC-based gel fuel droplets can be explained as follows. Prior to rupture, the viscoelastic shell restrains the internal pressure in a sequential manner by stretching, thinning, and yielding, and therefore, the nature of the jetting events is governed by the rheo-physical properties of the shell, such as its thickness, yield stress, and the strain that it undergoes before and after its yield point. While it is exceedingly difficult to measure these shell properties in a dynamic combusting environment, an estimate of the properties of the freshly prepared gel fuel can provide critical insights into the nature of the shell that these gellants would form. For instance, the yield stress of HPMC-3 is ~25 Pa, while that of MC-9 is ~400 Pa [35], which indicates that the MC-9 gel fuels would facilitate a stronger shell formation compared to HPMC-3 fuels. In addition, dynamic creep analysis in previous studies [19,35] showed that, at the same magnitude of applied shear stress below the yield stress values of both fuels, the strain endured by HPMC-3 was two orders of magnitude higher compared to the MC-9 gel fuel [19,35]. This means that HPMC-3 gel fuel droplets have a propensity to form weak-flexible shells, while the MC-9 gel fuel droplets tend to form a strong-rigid shell. A higher strength indicates that the shell will sustain higher pressures, while a high rigidity indicates that the shell will undergo minimal deformation/expansion prior to rupture. Such a strong-rigid shell tends to rupture with small, localized cracks through which the high pressure is released in the form of fire balls and pin hole jets, i.e., the jetting events that have nominally high velocities. In contrast, a weak-flexible shell indicates that it responds to pressure build-up by stretching and expansion, i.e., its flexibility. However, as the shell is weak compared to that of MC-9 gel fuels, it is unable to sustain very high pressures. Due to combined effects of lower internal pressures and a high shell flexibility, the HPMC-3 gel fuel droplets form larger rupture sites that result in low-velocity jets, which are typical of flame distortion jetting events. A qualitative comparison of the rupture sites in the HPMC-3 versus MC-9 gel fuels is shown in Figure 14.

## 3. Conclusions

Jetting is a critical phenomenon in the burning of gel fuel droplets that enables the efflux of the unreacted fuel vapors and, therefore, is likely to affect the local fuel–oxidizer ratio, gas phase mixing, and the droplet trajectory (due to the recoil thrust) in rocket engine environments. In this study, dual-mirror Z-type Schlieren imaging was used to investigate the jetting dynamics of burning gel fuel droplets at a temporal resolution of 0.33 ms and an optical resolution of 126.3 µm/pixel. The jetting behavior was investigated in ethanol gel fuels laden with two different types of organic gellants, namely, HPMC at 3 wt.% and MC at 9%. The following key conclusions are drawn from the study:

- The jetting of fuel vapors is responsible for the disruptive combustion behavior of gel fuel droplets and occurs in two potential modes, namely, either oscillatory bursting or isolated bursting, or both, where the latter features single or multiple jets initiating simultaneously.
- Both the jetting modes constitute jetting events which may either distort the flame front or form a fire ball outside the enclosed flame envelope or, alternatively, break the flame front locally and form a pin hole therein. Accordingly, these events are identified as flame distortion, fire ball, and pin hole jets, respectively. The pin hole jets are the highest-velocity jets (~1000–1550 mm/s) and, hence, apply a high local shear to the flame front, thereby causing its localized extinction, which appears as a break in the flame.
- The type of the gellant and the nature of the shell that it forms determine the types of jetting events that will dominate the combustion behavior of a gel fuel droplet.
- The gellants that tend to form thin-weak-flexible shells (HPMC at 3 wt.% in this study) are associated with a low degree of internal pressure build-up and the formation of large rupture sites, which result in low-velocity flame distortion jets (500–870 mm/s). In contrast, the gellants that form thick-strong-rigid shells (MC at 9 wt.% in this study) are associated with a high degree of internal pressure build-up and the formation of tiny, localized rupture sites, which result in high-velocity flame ball (800–1530 mm/s) and pin hole jets (1000–1550 mm/s).

While some models can predict the bursting of burning gel droplets, they cannot quantify the types of jetting events, their spatiotemporal evolution, and their interplay with the flame envelope. In this light, the jetting dynamics deciphered in this study using Schlieren imaging provide a fundamental understanding of the chaotic combustion behavior of organic-gellant-laden gel fuel droplets.

## 4. Materials and Methods

### 4.1. Materials

In this study, two fuel combinations were taken for the experiments. The gel fuels used were non-metalized ethanol-based fuels containing organic gellants. Gelled ethanol fuel is a tri-component fuel that consists of two different organic gellant combinations: (1) research-grade ethanol (99.8% pure; CAS No. 64-17-5) as the base fuel and (2) the gelling agents or gellants i.e., hydroxypropyl methylcellulose (HPMC; CAS No. 9004-67-3, $\rho_{B}\;\sim$ 689 kg/m^3^) and methylcellulose (MC; CAS No. 9004-67-5, bulk density $\rho_{B}\;\sim$ 504 kg/m^3^). MC has a methoxy group ranging from 27.5 to 31.5%, while HPMC has both active hydroxyl and methoxy groups ranging between 7–12% and 28–30%, respectively [35]. (3) Double-distilled water served as the base solvent for organic gellants. The properties and compositions of both fuel formulations are detailed in Table 2. Note that while the HPMC-based gels fuels were prepared over a range of concentrations from 3 to 6%, with a range of 8–9% for the MC-based fuels, for brevity, the results are presented for only two cases, because these fuels represent two extreme cases of jetting behavior.

During the preparation of the gel fuels, a key consideration was that the gel fuel should have maximum fuel content, together with a stable gel phase. In this regard, 3% of HPMC and 9% of MC fit this criterion. To obtain a stable gel fuel, three steps were employed. Firstly, the organic gellants (HPMC-3% or MC-9%) were added to ethanol and later subjected to manual stirring for ~2 min at room temperature. Secondly, the solvent, i.e., de-ionized water, was added to achieve a gel state. The gel formed after the addition of the solvent was subjected to mechanical stirring using a three-blade impeller at 500 rpm and for ~2 min. The resulting gel was then left undisturbed at room temperature for ~2 days. This resting period allows the 3D network formation process to be completed and helps to form a stable gel phase. Additionally, this period is utilized to perform a visual check for any phase separation that may occur during the network formation process and, therefore, allows one to analyze the time-dependent stability of the formulated gel fuel.

### 4.2. Experimental Test Facility

The combustion of the gel fuels was carried out in a pendant mode setup at ambient temperature, a normal pressure, and normal gravity conditions. A fixed volume of the droplet was extracted using a calibrated µ-syringe. The syringe dispensed ~2.8 µL of the gel. The suspension of the gel fuel droplet was performed using a fused quartz fiber of 80 µm. The quartz wire was chosen because its small size (diameter less than 100 µm) and low thermal conductivity (1.4 W/mK at 293 K) offer the least thermal and physical interference during combustion. The droplet ignition was achieved using a 150 µm nichrome wire via a DC power source. The droplet scale imaging was performed to record and observe the bursting dynamics associated with the combustion of the gel fuel droplets. For this purpose, an ultra-high-speed PHOTRON FASTCAM SA-X2 camera (Photron, Daejeon, Republic of Korea) with a 6.5× Navitar Zoom lens was used to capture high-speed videos at 10,000 fps with an exposure time of 60 µs at the spatial resolution of 3.9 µm/pixel. Due to the difficulties associated with the attainment of the spherical shape of the gel fuel droplet, the projected area of an equivalent sphere was taken. The average projected diameter of the HPMC-3% and MC-9% gel fuel droplets over six the experimental trials was ~1.65 ± 0.1 mm. The error in determining the droplet diameter was ±3%.

**Figure 15:**
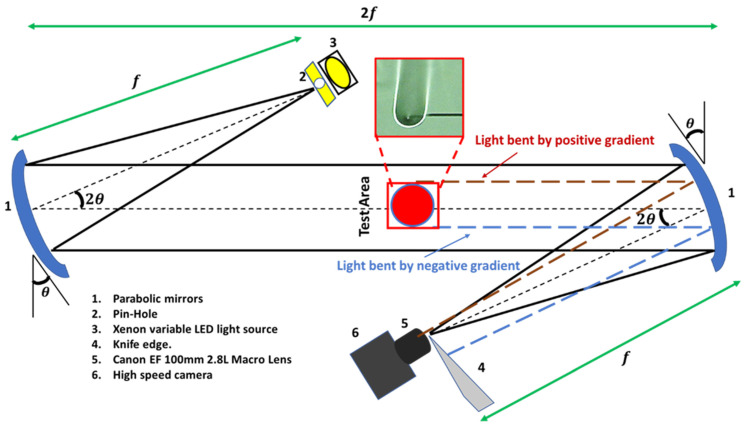
Schematic diagram of the experimental test facility showing the dual-mirror Z-type Schlieren system for characterizing the jetting behavior of the burning gel fuel droplets.

The flame scale imaging of both the gel fuels was performed using the Schlieren imaging technique [36], as shown in Figure 15. This setup uses an ultra-high-speed PHOTRON FASTCAM SA-X2 camera with a Canon EF 100 mm 2.8 L macro-lens to record the jetting dynamics. The flame data were recorded at 3000 fps at a spatial resolution of 126.3 µm/pixel.

The schlieren system consists of two parabolic mirrors (HOLMARC HO-SDIS-150) placed on gimble mounts, an LED point light source, a knife edge with an adjuster, and a high-speed camera. The gimble mounts of the parabolic mirrors eliminate the undesirable linear beam translation during the adjustment. The parabolic mirrors project the collimated beams of light rays onto the object under study to visualize the variations (or non-uniformities) in the refractive index that occur due to the changes in the air density, caused by changes in temperature and pressure. During imaging, a light or a darker area is created by the knife edge. The droplet flame is a diffusion flame with a high temperature along its periphery, which is surrounded by low-temperature regions, i.e., the inner region of the flame envelope and the outer surrounding air. This density difference appears as a bright (high-temperature) versus a dark region (low-temperature) in the Schlieren images. Furthermore, since the jets are composed of unreacted fuel vapors which have a different density compared to their surroundings, the Schlieren imaging technique can clearly track the jetting events and their interactions with the flame envelope. The velocities of the jets are calculated by measuring the jet as it travels from the droplet to the flame periphery divided by the travel time duration. The error in calculating the jet velocities occurs due to the difficulty in ascertaining the exact time when the jet impacts the flame envelope. This is because some jetting events occur in very short time scales of O ~ 5 ms.

### 4.3. Image Acquisition and Post-Processing: Flame Front Tracking and Reconstruction

**Figure 16:**
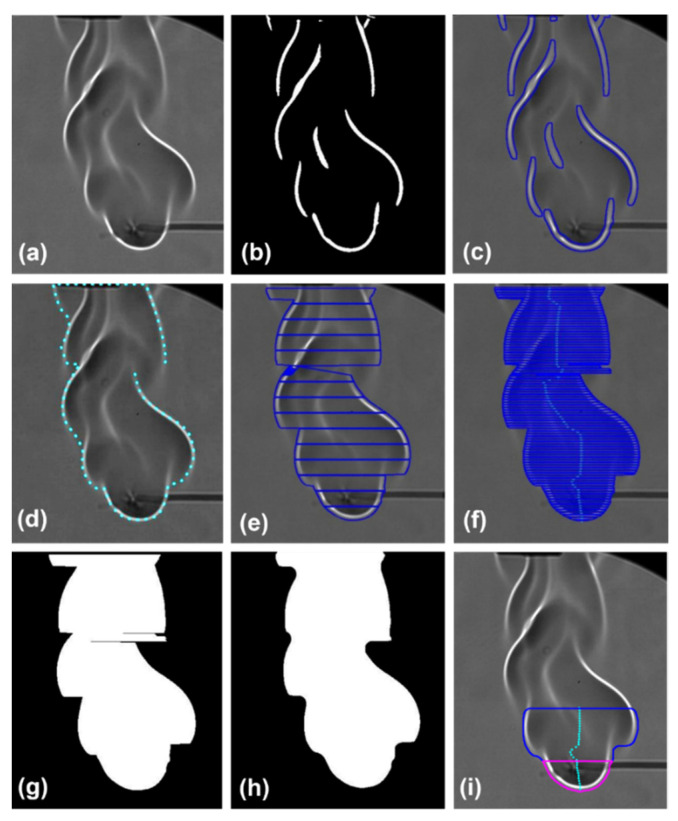
A sequential process of image processing for the extraction of the flame perimeter from the raw Schlieren images.

This section comprises the details regarding the flame-scale imaging of both sets of fuels, i.e., the ethanol-based HPMC-3% and MC-9% gel fuels. Deep learning is applied for flame tracking and calculations of the area and perimeter of the flame [37,38,39]. The Schlieren imaging setup produces a flame of a discontinuous periphery. This discontinuity illustrates the density gradients with respect to time. Consequently, to create a continuous and smooth flame, multiple frames of discontinuous flames are combined. Subsequently, the parameters such as flame perimeter and area are estimated throughout the flame combustion lifetime. The implementation of the algorithm is carried out in multiple steps, which are applied to each frame of the high-speed flame video. The first step is color thresholding, in which a localized area of the flame is taken and converted into a binary mask of the flame (Figure 16a,b). In the resulting binary image, the flame coordinates which surround the flame are extracted. The obtained coordinates are segregated based on the boundary and non-boundary points, of which the non-boundary points are later discarded. The boundary points are connected to form a uniform contour, which encircles the flame (Figure 16c,d). The uniform contour is obtained in steps. Initially, the flame is divided into horizontal sections of a fixed height (Figure 16e). These regions or sections are processed, as discussed above, and are later reconstructed to create a flame section (Figure 16f–h). Similarly, the entire flame is studied section-wise, and the sections are joined to form a silhouette of the flame. In the subsequent step, morphological transformations are utilized to obtain a smooth, refined, and perpetual flame boundary. The flame boundary is used for the calculation of the flame area and perimeter. These two parameters are computed for two regions to increase the accuracy of the extracted data (Figure 16i). In the region indicated in pink color, the flame standoff distance (SOD) from the droplet is measured, while the region in blue demarcates the area between the bounded region and the flame standoff distance, which is at distance between -SOD and 2 SOD.

The steps involved in the algorithm discussed thus far are shown in Figure 16. The details of the image processing algorithm are explained in the text in Supplementary Material (S1).

## Figures and Tables

**Table 1:** The magnitudes of the velocity of the three types of jetting events for both the gel fuels.

Flame Disruption Event	HPMC-3% (mm/s)	MC-9% (mm/s)
Flame distortion	800 ± 5%	873 ± 7%
Fireball outside the flame	1095 ± 10%	1030 ± 8%
Pin ejections	1313 ± 9%	1155 ± 6%

**Table 2:** Relative composition (in weight %) and yield stress of the test fuels.

Gellant	Weight %	Ethanol (Wt. %)	De-Ionized Water (Wt. %)	Yield Stress (Pa)
HPMC	3	82	15	23.23 ± 2.62
MC	9	77	14	398 ± 4.2

## Data Availability

Not applicable.

## Supplementary Materials

The following supporting information can be downloaded at: https://www.mdpi.com/article/10.3390/gels8120781/s1, S1: Flame front tracking and reconstruction.
