# Supplementary material for: Jetting Dynamics of Burning Gel Fuel Droplets
Source: Gels. 2022 Nov 29;8(12):781. doi: 10.3390/gels8120781 (PMC9777847; doi:10.3390/gels8120781)
Supplement: Supplementary file 1 [file gels-08-00781-s001.zip › gels-2032643-supplementary.pdf]

gels

MDPI

Article

Jetting Dynamics of Burning Gel Fuel Droplets

Janmejai Sharma [1], Ankur Miglani [1,*], Jerin John [2], Purushothaman Nandagopalan [3], Javed Shaikh [4] and Pavan Kumar Kankar [1]

[1] Microfluidics and Droplet Dynamics Lab, Department of Mechanical Engineering, Indian Institute of Technology, Indore 453552, Madhya Pradesh, India
[2] Department of Aerospace Engineering, Korea Advanced Institute of Science and Technology, 291, Daehak-ro, Yuseong-gu, Daejeon 34141, Republic of Korea
[3] Department of Aerospace Engineering, Hindustan University, Chennai 603103, Tamil Nadu, India
[4] Intel Corporation, Bengaluru 560035, Karnataka, India
* Correspondence: amiglani@iiti.ac.in

Supplementary Materials

S1. Flame front tracking and reconstruction.

Schlieren imaging produces high-contrast images that demarcate the regions based on the density gradients. However, due to the occurrence of vortex roll-up at the shear layer between the droplet flame envelope and its surrounding area and the occurrence of the jetting events, the symmetrical tear-drop shape of the flame is continuously disrupted. This results in flame images with a discontinuous periphery, thereby making it difficult to track and reconstruct the complete flame envelope, which is a prerequisite condition for calculating the flame area and perimeter. Therefore, a multi-step image-processing algorithm was developed, which combines the discontinuous entities across multiple images to generate a smooth and continuous flame envelope. This algorithm calculates the quantitative attributes of the flame envelope, i.e., the area and perimeter as a function of time, for any desired flame height.

Figure S1 shows the step-by-step method through which the image processing algorithm is implemented on individual frames for the tracking and reconstruction of the flame envelope.

First, color thresholding is applied to the images using a predetermined threshold value to localize the area being monitored and create its binary mask. The fact that the high-temperature (or low-density) flame boundary appears as a bright region and forms the flame periphery help us to determine the threshold value. The pixels with values lower than the threshold have an extremely low chance of being present on the flame boundary and, therefore, are set to zero. The resulting image (see Figure S1b) displays a mask of potential but discontinuous flame edges. In the second step, since the generated masks occupy a finite area (represented by the thickness of the flame envelope), the contours are constructed around the highlighted areas in the mask to extract the coordinates of a set of points that encapsulate these areas. The points on the outer edge of the contours are useful, as they represent boundary points; however, the points on the inner edge (non-boundary points) are redundant and, therefore, must be filtered. To achieve this, only the points corresponding to the minimum and maximum values of the x-coordinate are stored for each vertical location of the image, while the remaining ones are discarded. The final set of boundary points are shown in Figure S1d. Subsequently, the boundary points must be connected to form a uniform contour that encapsulates the flame. However, because the flame boundary is irregular, with multiple concave and convex curves, there is no preset criterion which provides the order in which the points must be joined to form a continuous contour. To enable this, the image is divided into horizontal sections (see Figure S1e) of a fixed small height such that the effects of the shape irregularities are eliminated, and the points in these regions are processed and reconstructed to create a section of the flame. In these horizontal sections, the points are sorted in a clockwise order using the centroid

Citation: Sharma, J.; Miglani, A.; John, J.; Nandagopalan, P.; Shaikh, J.; Kankar, P.K. Jetting Dynamics of Burning Gel Fuel Droplets. *Gels* **2022**, *8*, 781. https://doi.org/10.3390/gels8120781

Academic Editor: Aref Abbasi Moud

Received: 30 October 2022
Accepted: 22 November 2022
Published: 29 November 2022

Publisher's Note: MDPI stays neutral with regard to jurisdictional claims in published maps and institutional affiliations.

Copyright: © 2022 by the authors. Licensee MDPI, Basel, Switzerland. This article is an open access article distributed under the terms and conditions of the Creative Commons Attribution (CC BY) license (https://creativecommons.org/licenses/by/4.0/).

as the reference point, i.e., $(x_{min} + x_{max})/2$ at each height. These ordered points generate smaller sections within the flame, as shown in Figure S1f. While the horizontal sections are mostly accurate, some faulty cases may occur when the points are not detected. For instance, in Figure S1e, no points are detected on the flame boundary on the right-hand side. To resolve such cases, the points on the other side of the flame boundary in the given horizontal section are mirrored. The mirroring axes are considered as the vertical axis passing through the moving average of the centroid over five frames. The resolved flame sections are shown in Figure S1f, where the moving averages of the centroids are marked, and each horizontal section is ten pixels thick. Next, to join the horizontal sections to create a structured body, all the sections are drawn in a blank binary frame one after the other. The resulting image (see Figure S1g) nearly resembles the actual shape of the flame envelope, except for a few discontinuities. Therefore, to refine the shape of the flame envelope with smooth, rounded curves, morphological transformations are performed, with the original flame image and the circular kernel being used as inputs. The shape of the final flame envelope is obtained by performing two operations, namely, opening and closing. The opening operation involves erosion followed by the dilation of the binary image, which aids in the removal of the scattered noise from the image. The closing operation involves dilation followed by the erosion of the binary image. This helps to close the small holes inside the objects in the foreground and the small black points on the object. The final reconstructed image of the flame envelope obtained after the morphological transformations is shown in Figure S1g. The exact boundary of the flame envelope that is tracked and used to calculate the flame perimeter and the area is shown in Figure S1h. Note that in the area beyond the blue boundary, the vortex roll-up, due to KH instability [38], disturbs the flame shape and, therefore, is not considered in the calculation of the flame area in this study.

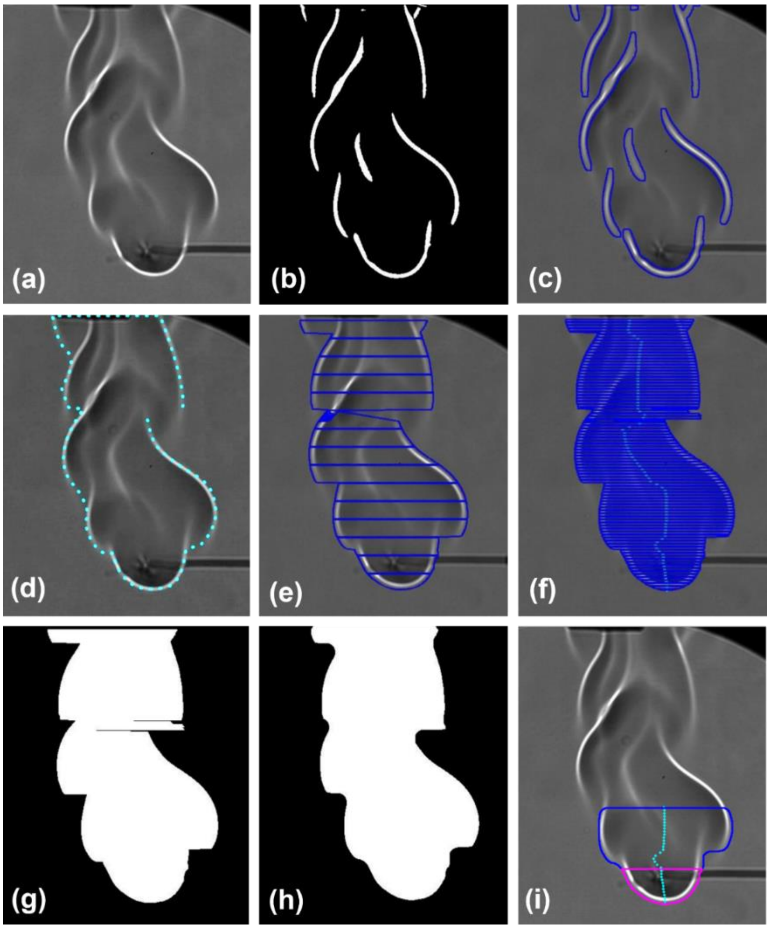

Figure S1. A sequential process of the image processing for extraction of the flame perimeter from the raw Schlieren images.
